# Leaves of Invasive Plants—Japanese, Bohemian and Giant Knotweed—The Promising New Source of Flavan-3-ols and Proanthocyanidins

**DOI:** 10.3390/plants9010118

**Published:** 2020-01-17

**Authors:** Maja Bensa, Vesna Glavnik, Irena Vovk

**Affiliations:** 1 Department of Food Chemistry, National Institute of Chemistry, Hajdrihova 19, SI-1000 Ljubljana, Slovenia; maja.bensa@gmail.com; 2 Faculty of Chemistry and Chemical Technology, University of Ljubljana, Večna pot 113, SI-1000 Ljubljana, Slovenia

**Keywords:** *Reynoutria*, *Polygonum*, *Polygonaceae*, flavanols, catechins, procyanidins, condensed tannins, HPTLC-MS, chemical profiling, fingerprints

## Abstract

This is the first report on identification of all B-type proanthocyanidins from monomers to decamers (monomers—flavan-3-ols, dimers, trimers, tetramers, pentamers, hexamers, heptamers, octamers, nonamers, and decamers) and some of their gallates in leaves of Japanese knotweed (*Fallopia japonica* Houtt.), giant knotweed (*Fallopia sachalinensis* F. Schmidt) and Bohemian knotweed (*Fallopia* × *bohemica* (Chrtek & Chrtkova) J.P. Bailey). Flavan-3-ols and proanthocyanidins were investigated using high performance thin-layer chromatography (HPTLC) coupled to densitometry, image analysis, and mass spectrometry (HPTLC–MS/MS). All species contained (−)-epicatechin and procyanidin B2, while (+)-catechin was only detected in Bohemian and giant knotweed. (−)-Epicatechin gallate, procyanidin B1 and procyanidin C1 was only confirmed in giant knotweed. Leaves of all three knotweeds have the same chemical profiles of proanthocyanidins with respect to the degree of polymerization but differ with respect to gallates. Therefore, chromatographic fingerprint profiles of proanthocyanidins enabled differentiation among leaves of studied knotweeds, and between Japanese knotweed leaves and rhizomes. Leaves of all three species proved to be a rich source of proanthocyanidins (based on the total peak areas), with the highest content in giant and the lowest in Japanese knotweed. The contents of monomers in Japanese, Bohemian and giant knotweed were 0.84 kg/t of dry weight (DW), 1.39 kg/t DW, 2.36 kg/t, respectively, while the contents of dimers were 0.99 kg/t DW, 1.40 kg/t, 2.06 kg/t, respectively. Giant knotweed leaves showed the highest variety of gallates (dimer gallates, dimer digallates, trimer gallates, tetramer gallates, pentamer gallates, and hexamer gallates), while only monomer gallates and dimer gallates were confirmed in Japanese knotweed and monomer gallates, dimer gallates, and dimer digallates were detected in leaves of Bohemian knotweed. The profile of the Bohemian knotweed clearly showed the traits inherited from Japanese and giant knotweed from which it originated.

## 1. Introduction

Japanese knotweed (*Fallopia japonica* Houtt.; synonyms: *Reynoutria japonica* Houtt., *Polygonum cuspidatum* Sieb. & Zucc., *Polygonum reynoutria* Makino), giant knotweed (*Fallopia sachalinensis* F. Schmidt; synonyms: *Reynoutria sachalinensis* (F. Schmidt) Nakai, *Polygonum sachalinense* F. Schmidt), and their interspecific hybrid Bohemian knotweed (*Fallopia* × *bohemica* (Chrtek & Chrtkova) J.P. Bailey; synonyms: *Reynoutria* x *bohemica* Chrtek & Chrtkova, *Polygonum* x *bohemicum* (Chrtek & Chrtkova) P.F. Zika & A.L. Jacobson) are perennial herbaceous plants that belong to the genus *Fallopia* and family *Polygonaceae* and are globally very problematic invasive alien plant species. Japanese knotweed was first brought to the Netherlands from Japan in the 19th century. Because Japanese and giant knotweed were popular as ornamental garden plants they quickly spread across Europe [1]. Today Japanese, giant and their hybrid Bohemian knotweed are widely spread in Europe and are considered to be alien invasive species. In the last two centuries, knotweeds grew and spread to the point of becoming an ecological and economical problem (endangering biodiversity and destroying infrastructure) in Europe, North America and also Australia and New Zealand. Japanese knotweed is on the list of the 100 most invasive alien species [2,3]. Among the reasons for the invasive nature of knotweeds is that they require very little to thrive. Hence, they are often found growing on riverbanks, at the edge of forests, near roads, on landfills, construction sites, railway embankments, and abandoned fields. Knotweeds spread with pieces of rhizomes, stems and seeds. In its native Japan Japanese knotweed grows on volcanic areas and lava fields and can be found growing even above the timberline at high altitudes on Mt. Fuji [1,3].

Since the attempts to eradicate these *Fallopia* species in Europe and North America have largely proved to be unsuccessful, efforts must be directed to their possible use. Fortunately, there is a great potential for the use of *Fallopia* knotweeds. Traditional Chinese medicine has been using Japanese knotweed in some medicines for treating different inflammatory diseases, diuretic problems, and even diarrhea [4]. There are food supplements with stilbene *trans*-resveratrol isolated from Japanese knotweed rhizomes. Different industries (e.g., food, paper) are researching the still untapped potential of knotweeds as a raw material for various purposes (e.g., a natural herbicide [5]).

Among Japanese, Bohemian and giant knotweed plant materials Japanese knotweed rhizomes were studied the most. Bioactive secondary metabolites from different groups like stilbenes [6,7,8,9], flavonoids [6,7,8,9], phenolic acids [6,7,8,9], carotenoids [9,10], chlorophylls [9] and triterpenic acids [8,9] were found in leaves of Japanese [6,7,8,9], Bohemian [10] and giant [8,9] knotweed.

Flavanols or flavan-3-ols or catechins represent the most structurally complex group of flavonoids. Flavan-3-ols can take monomer, oligomer or polymer forms which are also called condensed tannins or proanthocyanidins. One unit is constructed from a flavonoid skeleton C6-C3-C6 that are varied depending on stereochemistry and the number of hydroxyl groups. Flavan-3-ols have two chiral centers and thus four stereoisomers. Catechin is a monomer with *trans* configuration, while epicatechin has *cis* configuration. Proanthocyanidins are oligomers or polymers of flavan-3-ols. Proanthocyanidins are also sorted into subclasses according to their monomeric units and substitution patterns. One of proanthocyanidin subclasses are procyanidins with hydroxyl pattern 3, 3′, 4′, 5, 7 [11].

Recently, we tentatively identified flavan-3-ols and B-type proanthocyanidins from monomers up to decamers (monomers—flavan-3-ols, dimers, trimers, tetramers, pentamers, hexamers, heptamers, octamers, nonamers and decamers) and some of their gallates (monomer gallates, dimer gallate, dimer digallates, trimer gallates, tetramer gallates, pentamer gallates, hexamer gallates) in Japanese knotweed rhizomes [12,13]. Other authors reported identification of monomers [8,9,14,15,16], monomer gallates [8,14,16], dimers [8,9,15], dimer gallates [15,16] dimer digallates [15,16] trimers [15], trimer gallates [15] trimer digallates [15] tetramer [15], tetramer gallates [15], pentamers [15], heptamers [15] and octamers [15] in rhizomes of Japanese [8,9,14,15,16], Bohemian [14,15] and giant knotweed [8,9,14,15,16]. However, only monomers [8,9], monomer gallates [8,9], dimers [8,9], dimer gallates [8,9] and tetramers [8,9] of proanthocyanidins were detected in leaves [8,9] of Japanese knotweed [8,9] and giant knotweed [8,9]. To the best of our knowledge, no data is available for proanthocyanidins in leaves of Bohemian knotweed.

**Figure 1:**
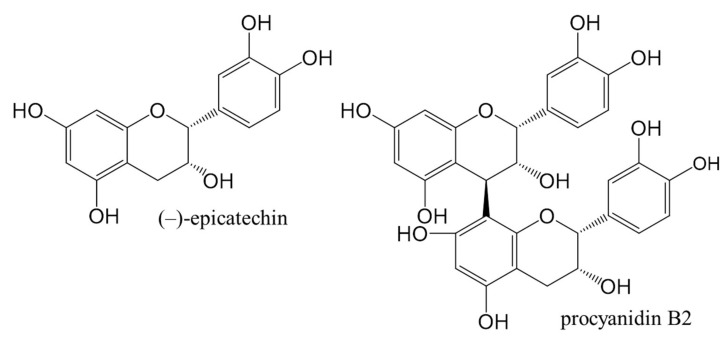
Chemical structures of (−)-epicatechin and procyanidin B2 standards used in this study.

The aim of our work was to: (i) obtain chromatographic fingerprints for test solutions from leaves of Japanese, Bohemian and giant knotweed; (ii) compare knotweeds’ total content of flavan-3-ol (monomers) calculated to standard (−)-epicatechin and the total dimer content calculated to standard procyanidin B2 (Figure 1); (iii) evaluate test solutions based on total peak area for proanthocyanidins; (iv) identify proanthocyanidins in the sample test solutions from leaves of Japanese, Bohemian and giant knotweeds using high performance thin-layer chromatography coupled to mass spectrometry (HPTLC-MS).

## 2. Results and Discussion

### 2.1. Qualitative Determination of Flavan-3-ols and Proanthocyanidins by HPTLC

All sample test solutions (STSs) prepared from leaves of Japanese, Bohemian and giant knotweed were analyzed using the HPTLC method, which we previously used for analyses of proanthocyanidins in Japanese knotweed rhizomes [12]. Separation was performed on HPTLC silica gel plate using toluene–acetone–formic acid (3:6:1, *v*/*v*) as a developing solvent in an unsaturated twin trough chamber followed by post-chromatographic derivatization with 4-dimethylaminocinnamaldehyde (DMACA) detection reagent [17]. Using DMACA reagent flavan-3-ols and proanthocyanidins were detected as blue-colored bands under visible light. The same combination of the stationary phase and developing solvent used for HPTLC-MS/MS analyses of Japanese knotweed rhizomes, which were performed on twice pre-developed plates, enabled a separation of proanthocyanidins according to their degree of polymerization [12].

**Figure 2:**
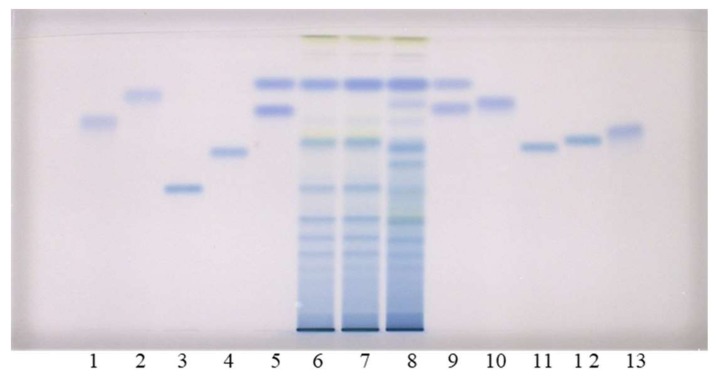
HPTLC chromatograms for the qualitative determination of flavan-3-ols and proanthocyanidins in STSs from leaves (2 μL, 50 mg/mL) of Japanese (track 6), Bohemian (track 7) and giant (track 8) knotweed based on standards of (−)-gallocatechin gallate (0.2 µg; track 1), (−)- catechin gallate (0.2 µg; track 2), procyanidin C1 (0.3 µg; track 3), procyanidin B3 (0.2 µg; track 4), (−)-epicatechin (0.1 µg; track 5, higher R_F_), (−)-epigallocatechin (0.2 µg; track 5, lower R_F_), (+)- catechin (0.1 µg; track 9, higher R_F_) (−)-gallocatechin (0.2 µg; track 9, lower R_F_), (−)-epicatechin gallate (0.2 µg; track 10), procyanidin B1 (0.2 µg; track 11), procyanidin B2 (0.2 µg; track 12) (−)- epigallocatechin gallate (0.2 µg; track 13). The HPTLC silica gel plate was developed with the developing solvent toluene-acetone-formic acid (3:6:1, *v*/*v*) and documented after derivatization with DMACA detection reagent at white light illumination.

**Figure 3:**
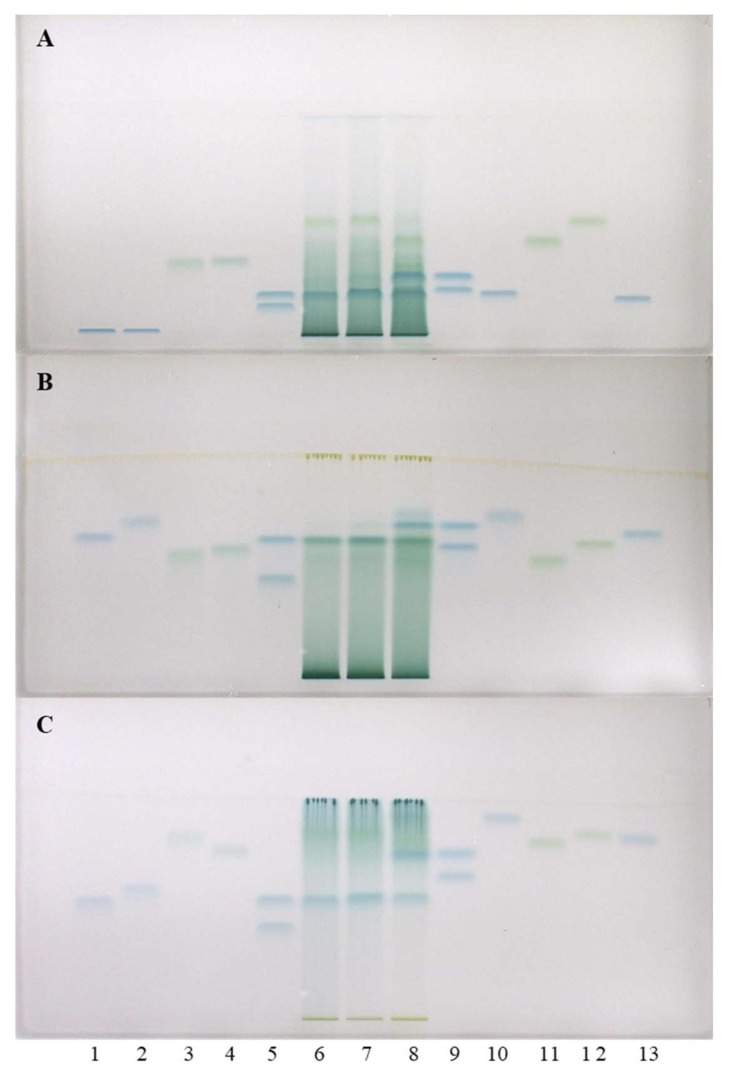
HPTLC chromatograms for the qualitative determination of flavan-3-ols and proanthocyanidins in STSs from leaves (1 μL, 50 mg/mL) of Japanese (track 6), Bohemian (track 7) and giant (track 8) knotweed based on standards of (−)-gallocatechin gallate (60 ng; track 1), (−)-catechin gallate (60 ng; track 2), procyanidin C1 (150 ng; track 3), procyanidin B3 (100 ng; track 4), (−)-epicatechin (50 ng; track 5, higher R_F_), (−)-epigallocatechin (60 ng; track 5, lower R_F_), (+)-catechin (50 ng; track 9, higher R_F_), (−)-gallocatechin (60 ng; track 9, lower R_F_), (−)-epicatechin gallate (60 ng; track 10), procyanidin B1 (120 ng; track 11), procyanidin B2 (90 ng, track 12) (−)-epigallocatechin gallate (60 ng; track 13). The HPTLC cellulose plates were developed with water (**A**), 1-propanol-water-acetic acid (4:2:1, *v*/*v*) (**B**), 1-propanol-water-acetic acid (20:80:1, *v*/*v*) (**C**) as developing solvents and documented after derivatization with DMACA detection reagent at white light illumination.

As can be seen in Figure 2, this method does not enable the separation of standards for monomers ((+)-catechin (track 9) and (−)-epicatechin (track 5)), and the separation of (−)-gallocatechin (track 9) and (−)-epigallocatechin (track 5). However, intensive blue bands in tracks of all STSs (tracks 6–8) from leaves at R_F_ of monomers ((+)-catechin and (−)-epicatechin) confirmed the presence of monomers in leaves of all studied knotweeds. Dimer procyanidin B2 (track 12) was detected based on the standard in STSs from all studied species, with intensive blue bands in tracks of STSs from Japanese (track 6, Figure 2) and Bohemian (track 7, Figure 2) knotweed and slightly less intensive bands in the track of STS from giant knotweed (track 8, Figure 2). (−)-Epicatechin gallate (track 10), dimer procyanidin B1 (track 11) and trimer procyanidin C1 (track 3) were detected based on the corresponding standards only in leaves of giant knotweed (Figure 2). Based on other applied standards (−)-catechin gallate (track 2), (−)-gallocatechin (track 9), (−)-epigallocatechin (track 5), (−)-epigallocatechin gallate (track 13) and dimer procyanidin B3 (track 4) were not detected in STSs from leaves (tracks 6–8). Due to the limitation of silica gel stationary phase in separation of some of the studied compounds, further examinations were performed on HPTLC cellulose plates (Figure 3), which were developed based on the previously developed methodology [17,18,19], which provided complementary results.

Separations on HPTLC cellulose plates were performed with three developing solvents (water [17,18,19], 1-propanol–water–acetic acid (4:2:1, *v*/*v*) [19] and 1-propanol-water-acetic acid (20:80:1, *v*/*v*) [19]) and detection was performed with DMACA reagent. As can be seen in Figure 3, (−)-epicatechin (track 5) and procyanidin B2 (track 12) were detected in Japanese (track 6), Bohemian (track 7) and giant (track 8) knotweed leaves, while (−)-gallocatechin (track 9), (−)-epigallocatechin (track 5) and (−)-epigallocatechin gallate (track 13) were not detected in any STSs. These results were confirmed on HPTLC cellulose stationary phase with all three developing solvents. Additionally, (+)-catechin (track 9) was detected in leaves of Bohemian (track 7) and giant (track 8) knotweed on HPTLC cellulose plates with all applied developing solvents (Figure 3). Using the corresponding standards (−)-epicatechin gallate (track 10), dimer procyanidin B1 (track 11) and trimer procyanidin C1 (track 3) were detected only in leaves of giant knotweed (track 8) and were confirmed on HPTLC cellulose stationary phase with all three developing solvents (Figure 3).

The results obtained with all chromatographic systems that involved two HPTLC silica gel and HPTLC cellulose stationary phases in combination with all applied developing solvents (toluene-acetone-formic acid (3:6:1, *v*/*v*) for silica gel plates and water as well as 1-propanol-water-acetic acid in different ratios (4:2:1, *v*/*v*; 20:80:1, *v*/*v*)) are summarized in Table 1. It is evident that complementary methods are crucial to avoid reporting false positive results. Although different combinations of the stationary phase and the developing solvent resulted in certain cases in different results, the final confirmation of compounds was only awarded to those which were detected using both stationary phases (column “Both” in Table 1) and all developing solvents.

To the best of our knowledge this is the first report on detection of: (i) procyanidin B2 in Japanese knotweed leaves; (ii) (+)-catechin, (−)-epicatechin and procyanidin B2 in Bohemian knotweed leaves; iii) (−)-epicatechin gallate, dimer procyanidin B1 and trimer procyanidin C1 in giant knotweed leaves.

### 2.2. Chromatographic Fingerprinting (HPTLC) of Proanthocyanidins with Densitometry and Image Analysis

In Guidelines for the assessment of herbal medicines from 1991 WHO suggests chromatographic fingerprint for identification of a characteristic substance or mixture of substances when ensuring the consistent quality of plant preparations [20]. In addition, since 2004 the State Food and Drug Administration of China (SFDA) requires standardization by chromatographic fingerprints for all the injections made from herbal medicines or their raw materials [21]. Therefore, chromatographic fingerprinting of plant materials is often used as one of the key approaches of quality control of medicinal plants and plant materials used in the production of food supplements and different herbal (medicinal plants) preparations. Combined with different chemometric methods chromatographic fingerprinting is also used in studies of plant material [22] and for confirmation of the authenticity of propolis [23].

In this study chromatographic fingerprinting of proanthocyanidins was performed with densitometry before and after post-chromatographic derivatization with DMACA reagent. Due to technical limitations of the DigiStore 2 documentation system (only permitting wavelengths 366 nm and 254 nm to be used in UV zone) chromatographic fingerprinting was only done after post-chromatographic derivatization at white light.

**Figure 4:**
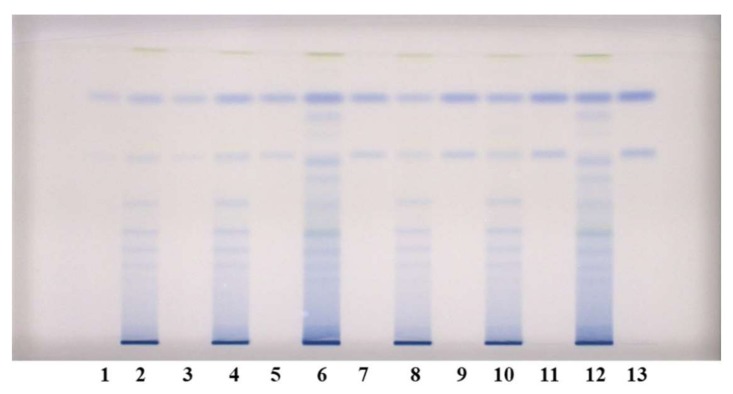
HPTLC chromatograms used for quantitative determination of proanthocyanidins in STSs from leaves (1 μL, 50 mg/mL) of Japanese (tracks 2 and 8), Bohemian (tracks 4 and 10) and giant (tracks 6 and 12) knotweed and standard solutions of (−)-epicatechin and procyanidin B2. The HPTLC silica gel plate was developed with the developing solvent toluene–acetone–formic acid (3:6:1, *v*/*v*) and documented after derivatization with DMACA detection reagent at white light illumination. Tracks of (−)-epicatechin and procyanidin B2 standard solutions: 1. 30 ng; 3. 40 ng: 5. 60 ng; 7. 80 ng; 9. 100 ng; 11. 120 ng; 13. 150 ng.

**Figure 5:**
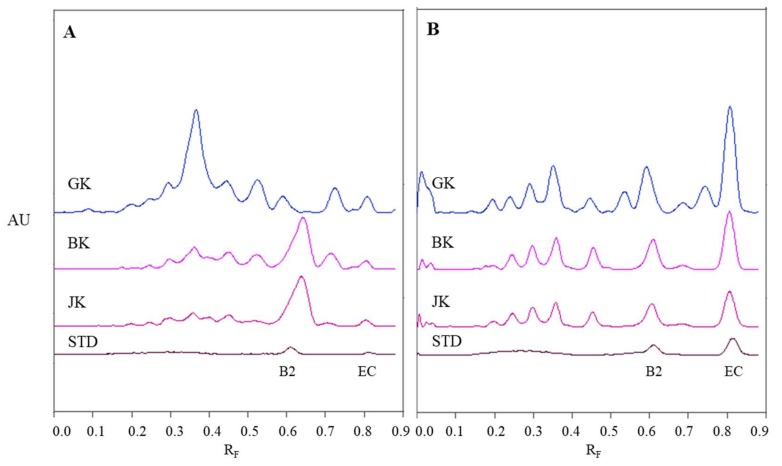
Densitograms of STSs (1 μL, 50 mg/mL) from leaves of Japanese (**JK**), giant (**GK**) and Bohemian (**BK**) knotweed and standard solutions (**STD**, 40 ng) of (−)-epicatechin (**EC**) and procyanidin B2 (**B2**) scanned in absorption/reflectance mode at 280 nm before derivatization (**A**) and at 655 nm after derivatization with DMACA reagent (**B**). HPTLC silica gel plate was developed with toluene-acetone-formic acid (3:6:1, *v*/*v*).

Chromatographic fingerprints of proanthocyanidins in STSs from leaves of Japanese, Bohemian and giant knotweed were firstly obtained by densitometric scanning of the HPTLC silica gel plates after development and then also after post-chromatographic derivatization with DMACA reagent (Figure 4). Densitometric scanning was performed in the absorption/reflectance mode at two wavelengths 280 nm (Figure 5A) and 655 nm (Figure 5B) to detect underivatized proanthocyanidins and their derivatives (formed when proanthocyanidins react with DMACA reagent), respectively.

The densitograms of (−)-epicatechin and procyanidin B2 show that derivatization with DMACA reagent drastically enhanced the sensitivity of the HPTLC method which is seen from the considerable increase in heights of peaks for both standards after derivatization (Figure 5B). The increase of peak heights is also seen in densitograms of STSs at R_F_ values for monomers (R_F_ of (−)-epicatechin) and dimers (R_F_ of procyanidin B2) of B-type proanthocyanidins. In Figure 5B we can see that leaves of Japanese, Bohemian and giant knotweed contain monomers and dimers of B-type proanthocyanidins, because the densitograms of all STSs contain two peaks at the same R_F_ values as the standard solutions of (−)-epicatechin and procyanidin B2. There are quite a few differences between chromatographic fingerprints of STSs from leaves (Figure 5A and Figure 4B) recorded on the same plates before and after derivatization. This is due to the fact that at 280 nm the light is absorbed not only by flavan-3-ols and proanthocyanidins, but also by other types of compounds (e.g., phenolic acids, other flavonoids) that are also present in knotweed STSs. Therefore, peaks at other R_F_ values are also visible.

In order to perform chromatographic fingerprinting by analysing images with the DigiStore 2 documentation system, the images of the HPTLC plates captured after post-chromatographic derivatization at white light illumination were converted to a different format using WinCATS software (Camag) and then converted to videodensitograms in absorption mode using VideoScan software (Camag).

**Figure 6:**
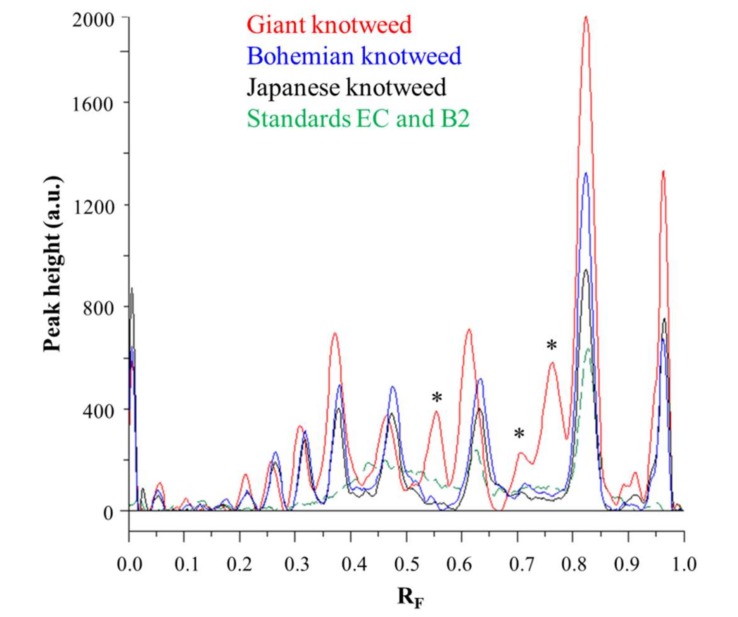
Comparison of videodensitogram fingerprint profiles of STSs (1 μL, 50 mg/mL) from leaves of Japanese, Bohemian and giant knotweed with videodensitogram of standards (−)-epicatechin (**EC**; R_F_ = 0.82) and procyanidin B2 (**B2**; R_F_ = 0.63) (30 ng; dashed green line). Videodensitograms were obtained in absorption mode by images analysis of HPTLC silica gel plates after development with developing solvent toluene–acetone–formic acid (3:6:1, *v*/*v*) and after derivatization with DMACA detection reagent. Asterisks (*) indicate peaks that are specific to giant knotweed leaves.

Videodensitograms of leaves STSs of all three knotweeds show peaks at R_F_ values of (−)-epicatechin and procyanidin B2 standards, which confirms the presence of monomers and dimers in all knotweed STSs (Figure 6). Comparison of videodensitograms of all STSs showed the highest variety in the qualitative proanthocyanidin profile for the STS from leaves of giant knotweed. Equal qualitative profiles were observed for the videodensitograms of the STSs from leaves of Japanese and Bohemian knotweeds, while three additional peaks at R_F_ 0.56, 0.71, and 0.76 are present in the giant knotweed profile (Figure 6). Additionally, from the peak heights in videodensitograms of all STSs it is evident that giant knotweed is the richest source of compounds presented with all the peaks.

### 2.3. Quantification of Proanthocyanidins

We investigated the differences in monomer and dimer content in leaves of all three knotweeds and which of these samples has the highest total proanthocyanidins content. Quantitative determination of proanthocyanidins was performed using HPTLC combined with image analysis. Testing of stability of derivatized standards of (−)-epicatechin (100 ng) and procyanidin B2 (100 ng) by image analysis (at 0, 5, 10, 15 and 20 min subsequently) showed no statistically significant differences at the 95% confidence level (*t*-test) in the mean peak areas. Limits of detection (LOD) on HPTLC silica gel plate were 5 ng and 15 ng, while limits of quantification (LOQ) were 10 ng and 20 ng for (−)-epicatechin and procyanidin B2, respectively. Analyses of STSs from leaves were performed on HPTLC silica gel plate (Figure 4) using equal amounts of (−)-epicatechin and procyanidin B2 standards (30, 40, 60, 80, 100, 120 and 150 ng per plate) for calibration curves. Videodensitograms were integrated using VideoScan software. Using peak area, we calculated the calibration curves for each standard. According to the established use of the data-pair technique in quantitative HPTLC analysis we calculated the peak areas mean value (at the same R_F_) for pairs of equal applications for each STS from the left and right side of the plate and by this reduced the effect of inequity of the stationary phase. Using the calibration curves equations for (−)-epicatechin and procyanidin B2 we calculated the contents of monomers (flavan-3-ols) and procyanidin dimers, respectively, in leaves of Japanese, Bohemian and giant knotweed (Table 2). The contents of monomers and dimers expressed per dry weight (DW) of the plant material for all STSs were the highest in leaves of giant knotweed and the lowest in leaves of Japanese knotweed (Table 2). This is the first report about quantification of total content of monomers and dimers proanthocyanidins in leaves of Japanese, Bohemian and giant knotweed. Additionally, this is the first report on proanthocyanidin analysis in leaves of Bohemian knotweed.

**Figure 7:**
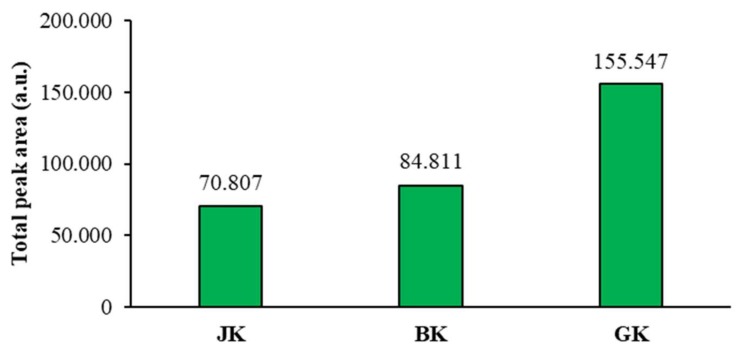
Comparison of the mean of the total peak areas of proanthocyanidins (blue bands in chromatograms) for STSs from leaves of Japanese (**JK**), Bohemian (**BK**), and giant (**GK**) knotweed. The mean of the total peak areas was calculated from the total peak areas of videodensitograms of two equal applications of the same STS on HPTLC silica gel plates (Figure 4) after development with developing solvent toluene-acetone-formic acid (3:6:1, *v*/*v*) and after derivatization with DMACA detection reagent.

The mean of the total peak areas of proanthocyanidins (all blue bands in chromatograms) calculated from the total peak areas of videodensitograms of two equal applications of the same STS on HPTLC silica gel plates offered additional information (Figure 7) about proanthocyanidins in all STSs. The comparison of the mean values of the total peak areas of proanthocyanidins proved the highest contents of proanthocyanidins in giant knotweed and the lowest in Japanese knotweed (Figure 7).

### 2.4. HPTLC-MS/MS Characterisation of Flavan-3-ols and Proanthocyanidins on Diol Stationary Phase

Using different HPTLC-MS/MS methods based on the separations performed on the HPTLC silica gel plates, we were the first to identify proanthocyanidins from monomers to decamers (of B-type procyanidins) in rhizomes of Japanese knotweed [12]. Recently, we developed additional HPTLC and HPTLC-MS/MS methods with separations on HPTLC diol F_254S_ plates [13], which enabled us to identify the above-mentioned compounds in the same plant material. The last methods enable faster analysis time, longer developing distances and therefore, better resolution, as well as less time-consuming pre-development step, which can even be avoided.

**Figure 8:**
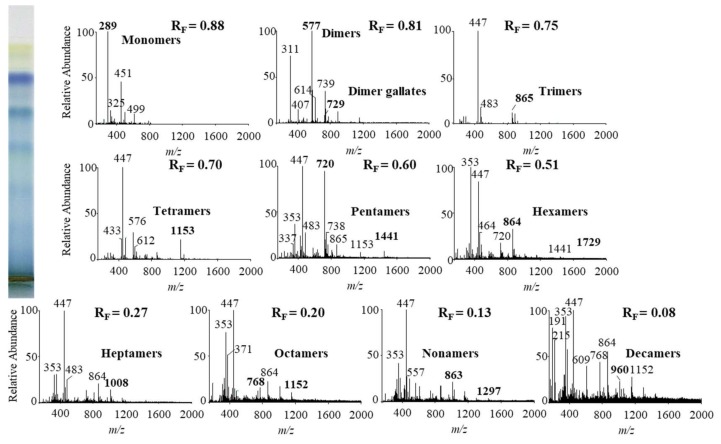
MS spectra obtained by HPTLC-MS analyses of STS from Japanese knotweed leaves on HPTLC diol F_254S_ plate pre-developed and developed with acetonitrile. The bolded *m/z* values in the MS spectra belong to B-type proanthocyanidins and their gallates, which were eluted from the plate with acetonitrile–methanol (2:1, *v*/*v*). A part of the plate was derivatized with DMACA reagent.

**Figure 9:**
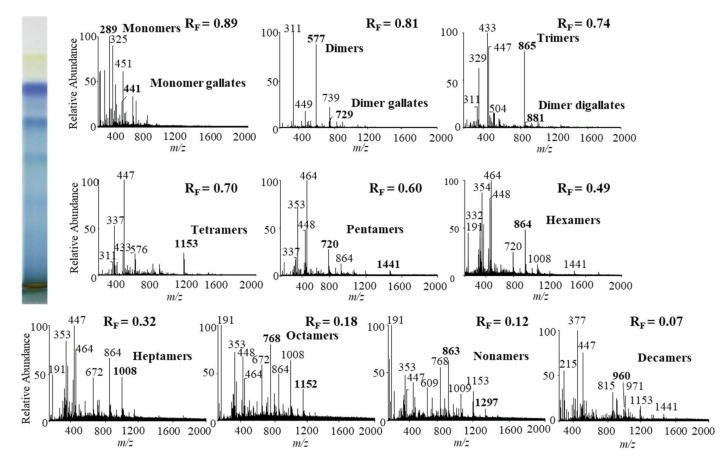
MS spectra obtained by HPTLC-MS analysis of STS from Bohemian knotweed leaves on HPTLC diol F_254S_ plate pre-developed and developed with acetonitrile. The bolded *m/z* values in the MS spectra belong to B-type proanthocyanidins and their gallates, which were eluted from the plate with acetonitrile–methanol (2:1, *v*/*v*). A part of the plate was derivatized with DMACA reagent.

**Figure 10:**
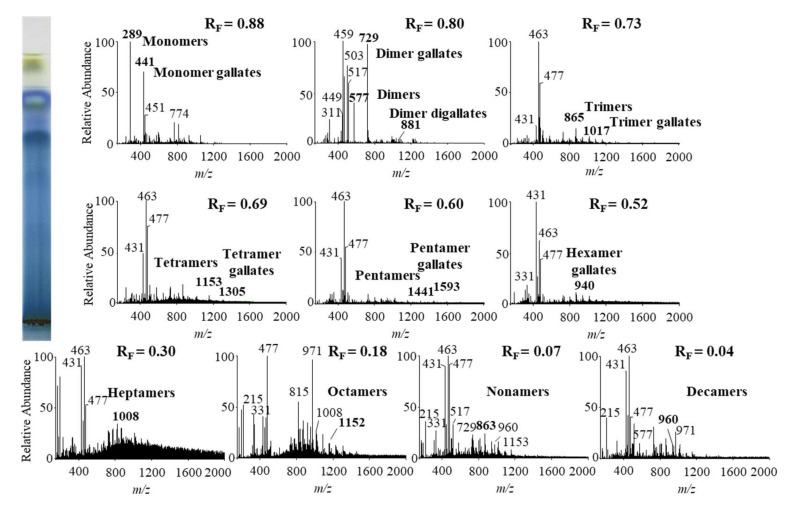
MS spectra obtained by HPTLC–MS analysis of STS from giant knotweed leaves on HPTLC diol F_254S_ plate pre-developed and developed with acetonitrile. The bolded *m/z* values in the MS spectra belong to B-type proanthocyanidins and their gallates, which were eluted from the plate with acetonitrile–methanol (2:1, *v*/*v*). A part of the plate was derivatized with DMACA reagent.

In this study we performed HPTLC-MS/MS analyses of proanthocyanidins in STSs from Japanese, Bohemian and giant knotweed leaves on HPTLC diol F_254S_ plates pre-developed and developed with acetonitrile. Equal volumes (150 μL) of STSs (50 mg/mL) prepared from leaves of Japanese, Bohemian and giant knotweed leaves were applied on separate plates. Since after development proanthocyanidins bands were not visible on the plate, the left incised (but not cut) edge of each plate was broken off and derivatized with DMACA reagent. With this approach, the analytes on the underivatized part of the plate were not exposed to possible degradation (due to heating) before detection with MS. The obtained blue-colored proanthocyanidins zones on the derivatized part of the plate were then used for proper positioning of elution head of TLC-MS interface on the parallel zones with the same R_F_ on the underivatized part of the plate, which were eluted from the plate and transferred to the MS detector for acquisition of the MS, MS^2^ and MS^3^ spectra. Due to the lack of available standards only monomers, monomer gallates and procyanidin dimers were identified by comparison of the fragmentation pattern with those of commercial standards (+)-catechin, (−)-epicatechin, (−)-epicatechin gallate, (−)-epigallocatechin gallate, (−)-epigallocatechin and procyanidin B2, respectively. Oligomers with higher degree of polymerisation (trimers to decamers), their gallates and digallates were tentatively identified primarily based on their MS spectra (Table 3, Figure 8, Figure 9 and Figure 10). These compounds were then additionally confirmed by MS^2^ and MS^3^ spectra and by comparison of obtained and published fragmentation patterns. Due to the limitations of mass range of MS detector proanthocyanidins with molecular mass greater than 2000 were identified as double- or triple-charged deprotonated molecular ions.

The results of HPTLC-MS/MS analyses of STSs from leaves of Japanese (Figure 8), Bohemian (Figure 9) and giant (Figure 10) knotweed (Table 3) were compared with the results of our previous analyses of STSs of Japanese knotweed rhizomes [12,13], performed using the same HPTLC–MS/MS method [13] and HPTLC-MS/MS method performed on HPTLC silica gel plates [12].

Literature data about proanthocyanidins identified (by using mass spectrometry) in leaves of knotweeds are rather scarce. The two available publications provide only data about monomers, monomer gallate, B-type procyanidin dimers, tetramers and dimer gallate identified in leaves of Japanese and giant knotweed [8,9]. So, this study is the first to report identification of proanthocyanidins in leaves of Bohemian knotweed. Additionally, we were the first to identify all B-type proanthocyanidins from monomers to decamers (monomers, dimers, trimers, tetramers, pentamers, hexamers, heptamers, octamers, nonamers and decamers), as well as some of their gallates in leaves of Japanese, Bohemian and giant knotweed (Table 3). We identified monomer gallates, dimer gallates, dimer digallates, trimer gallates, tetramer gallates, pentamer gallates and hexamer gallates in STSs from giant knotweed leaves (Table 3). Among all investigated STSs from leaves the highest variety in qualitative profile of gallates was found in STS from giant knotweed leaves. The results confirmed (Table 3) that STS from giant knotweed leaves had the same qualitative profile of gallates, ranging from monomer gallates to hexamer gallates and also dimer digallate, as were reported in our previous studies for Japanese knotweed rhizomes [12,13]. Only monomer gallate and dimer gallate were identified in STSs of Japanese knotweed leaves, while monomer gallate, dimer gallates, and dimer digallates were identified in STSs of Bohemian knotweed leaves (Table 3). This is the first report on dimer digallates confirmed in leaves Bohemian and giant knotweed. Based on this, we can conclude that leaves of Japanese, Bohemian and giant knotweeds have the same qualitative chemical profiles of proanthocyanidins concerning the degree of polymerization (Table 3), what is more the profiles are also the same as the profiles of Japanese knotweed rhizomes analyzed in our previous study [12,13], whereas the qualitative chemical profiles of proanthocyanidins concerning gallates differ among leaves of all three studied knotweeds. Additionally, we can conclude that within the individual species, Japanese knotweed leaves and rhizomes have equal qualitative chemical profiles of proanthocyanidins concerning the degree of polymerization, while concerning the profile of gallates rhizomes possess higher variety than leaves.

Other high intensity peaks in the MS spectra, which we did not identify as this was not the focus of our experiments, also appeared in the mass spectra. Nevertheless, based on the conditions of chromatography, elution into the mass spectrometer and findings from the literature it can be assumed that the remaining more intense peaks are probably mainly flavonoids and phenolic acids.

## 3. Materials and Methods

### 3.1. Chemicals

All chemicals were at least of analytical grade. Toluene, 1-propanol, formic acid, acetic acid, hydrochloric acid (37%) and 4-dimethylaminocinnamaldehyde (DMACA) were from Merck (Darmstadt, Germany). Ethanol, acetone, as well as HPLC grade acetonitrile and methanol were purchased from Sigma-Aldrich (St. Louis, MO, USA). LC-MS grade acetonitrile and methanol used for MS analyses were from Fluka (Buchs, Switzerland). MilliQ 18.2 MΩ water (Millipore, Bedford, MA, USA) was also used.

Standards of (−)-epicatechin gallate, (−)-epigallocatechin, (−)-epigallocatechin gallate, (−)-catechin gallate, (−)-gallocatechin, (−)-gallocatechin gallate, procyanidin B1, procyanidin B2, procyanidin B3 and procyanidin C1 were obtained from Extrasynthèse (Genay, France), while (−)-epicatechin was from Sigma-Aldrich and (+)-catechin from Carl Roth (Karlsruhe, Germany).

### 3.2. Preparation of Standard Solutions

Stock solutions of standards (0.1 mg/mL) were individually prepared in methanol. Separate working solutions (10 μg/mL) were prepared by dilution of stock solutions with methanol. All standard solutions were stored in amber glass storage vials at −80 °C.

### 3.3. Plant Materials

Leaves of Japanese knotweed (*Fallopia japonica* Houtt.), Bohemian knotweed (*Fallopia* x *bohemica* (Chrtek & Chrtkova) J.P. Bailey) and giant knotweed *(Fallopia sachalinensis* F. Schmidt) collected in Ljubljana (Slovenia) at the beginning of September 2018. The plants were in phenophase 61 (according to BBCH scale) — the beginning of flowering.

Leaves were frozen with liquid nitrogen and lyophilized (Micro Modulyo, IMAEdwards, Bologna, Italy) for 48 h at −50 °C. Lyophilized materials of all three samples were again individually frozen with liquid nitrogen before they were crushed and pulverized with Mikro-Dismembrator S (Sartorius, Göttingen, Germany) at a frequency of 1700 min^−1^ for 1 min. All lyophilized powdered materials were stored at −20 °C until they were used for the preparation of sample test solutions (STSs) of each plant material.

### 3.4. Preparation of Sample Test Solutions (STSs) From Leaves

STSs (50 mg/mL) from leaves were prepared separately by dispersing 200 mg of powdered lyophilized plant material in 4 mL of 70% aqueous acetone followed. Suspension was vortexed (1 min at 2800 rpm; IKA lab dancer, Sigma-Aldrich) and then centrifuged at 4500 rpm (Centric 322 A, Tehtnica, Železniki, Slovenia) for 5 min. Supernatant was filtered through a 0.45 µm polyvinylidene fluoride (PVDF) membrane filter (Millipore, Billerica, MA, USA). The obtained supernatants were stored in an amber glass storage vial at −20 °C. Due to smaller particles that appeared on the bottom of some STSs vials after thawing, those solutions were centrifuged for 3 min at 13,400 rpm (MiniSpin centrifuge, MiniSpin, Eppendorf, Hamburg, Germany). Supernatants (STSs from leaves of all knotweeds) were transferred into GC vials and were used undiluted for HPTLC and HPTLC-MS/MS analyses.

### 3.5. HPTLC with Image Analysis and Densitometry

HPTLC analyses of flavan-3-ols and proanthocyanidins were performed on unpre-developed 20 cm × 10 cm glass backed HPTLC silica gel plates (Merck, Art. No. 1.05641) and HPTLC cellulose plates (Merck, Art. No. 1.05786). STSs and standards solutions were applied on the plates by an automatic TLC Sampler 4 (Camag, Muttenz, Switzerland) as 10 mm bands, 8 mm from the bottom of the plate and 25 mm from the left edge. Different application volumes of STSs (50 mg/mL) and solutions of standards (0.01 mg/mL; (−)-epicatechin, (+)-catechin, (−)-epicatechin gallate, (−)-epigallocatechin gallate, (−)-epigallocatechin, (−)-catechin gallate, (−)-gallocatechin, (−)-gallocatechin gallate, procyanidin B1, procyanidin B2, procyanidin B3 and procyanidin C1) were used for qualitative analysis on HPTLC silica gel plate and HPTLC cellulose plates. Lower volumes of STSs (1 μL; 50 mg/mL) and the solutions of (−)-epicatechin and procyanidin B2 standards (3, 4, 6, 8, 10, 12 and 15 µL; 0.01 mg/mL) were applied on the HPTLC silica gel plate used for chromatographic fingerprinting and quantitative analyses. Each STSs from leaves was applied on the plate twice using data-pair technique (one application on the left and the other on the right half of the plate). All HPTLC silica gel plates were developed up to 9 cm using 10 mL of a developing solvent toluene-acetone-formic acid (3:6:1, *v*/*v*) [12], which was added only in one trough of an unsaturated twin-trough chamber (Camag) for 20 cm x 10 cm plates. Developing time was 25 min. All HPTLC cellulose plates were developed in a horizontal developing chamber (sandwich configuration; Camag) for 20 cm x 10 cm plates using the following developing solvents: water [17,18,19], 1-propanol–water–acetic acid (4:2:1, *v*/*v*) [19] and 1-propanol–water–acetic acid (20:80:1, *v*/*v*) [19].

After development and drying in a stream of warm air for 1 min, post-chromatographic derivatization was performed by dipping the plates for 1 s into DMACA dipping detection reagent prepared by dissolving 60 mg of DMACA in 13 mL of concentrated hydrochloric acid, which was made up to 200 mL with ethanol [17]. Dipping was followed by drying for 2 min in a stream of warm air.

Images of the plates were captured immediately after development and also 10 min after post-chromatographic derivatization, with a DigiStore 2 Documentation system (Camag) at 366 nm and white light illumination. For the purpose of image analyses, the obtained images of the HPTLC plates captured at white light illumination after post-chromatographic derivatization were converted to a different format using WinCATS software and then converted to videodensitograms in absorption mode using VideoScan TLC/HPTLC Evaluation Software (Version 1.02.00) (Camag). Densitometric analyses were performed by slit-scanning densitometer TLC Scanner 3 (Camag) set in the absorption/reflectance mode at 280 nm (before derivatization) or 655 nm (10 min after derivatization). The slit dimensions were: length 6 mm, width 0.3 mm; and the scanning speed 20 mm/s. All instruments were controlled by the winCATS software (Version 1.4.9.2001).

### 3.6. HPTLC-MS/MS Analyses

HPTLC-MS/MS analyses were performed on HPTLC glass backed diol F_254S_ plates (20 cm × 10 cm, Merck, Art. No. 1.05636), which were pre-developed with acetonitrile [13], dried for 5 min in a stream of warm air and cut in 10 cm × 10 cm parts. Plates were incised (but not cut) 15 mm from the left edge before application of STSs by Linomat 5 (Camag) as 60 mm bands, 8 mm from the bottom of the plate. STSs (150 μL; 50 mg/mL), prepared from leaves of Japanese, Bohemian and giant knotweed, were analyzed on separate plates. Plates were developed up to 9 cm with acetonitrile [13] in an unsaturated twin trough chamber (Camag) for 10 cm × 10 cm plates in 15 min. After development and drying for 1 min in a stream of warm air, the leftmost 15 mm part was broken off from the plate and only this part was dipped for 1 s into DMACA detection reagent for proanthocyanidin visualization (blue zones). Ten minutes after drying in a stream of warm air the derivatized part of the plate was attached to the underivatized part of the plate with scotch tape and documented by DigiStore 2 Documentation system. The blue zones were used for proper positioning of elution head of TLC-MS interface (Camag) on the parallel zones with same R_F_ in the remaining part of the track on the underivatized part of the plate, which were eluted from the plate and transferred to the MS detector for acquisition of first-order mass (MS), product ion (MS^2^) and (MS^3^) spectra.

A quaternary pump Accela Pump (part of the UHPLC system, Thermo Fisher Scientific, Waltham, MA, USA) and a TLC-MS interface with an oval elution head (4 mm × 2 mm) were used for on-line elution of analytes from the developed HPTLC diol F_254S_ plates into a LTQ Velos mass spectrometer with dual-pressure linear ion trap mass analyzer (Thermo Fisher Scientific). Acetonitrile–methanol (2:1, *v*/*v*) [12,13,24] at a flow rate of 0.2 mL/min was used as an eluent. An in-line filter (Idex, Health & Science, Oak Harbor, WA, USA) was installed between the TLC-MS interface and the ion source. Heated electrospray ionization (HESI) ion source in negative ion mode was used for ionization of compounds. The MS capillary temperature and heater temperature were 350 °C and 250 °C, respectively. Other MS conditions were as follows: sheath gas 35 a.u. (arbitrary units), auxiliary gas 10 a.u., sweep gas 0 a.u., spray voltage 3.5 kV, S-lens RF level 65% and capillary voltage −38.8 V. MS spectra were acquired for 1 min in the *m/z* range 150–2000. Fragmentation of the parent ion was performed at 30% collision energy and isolation width of 1.0 *m/z*. The collected data were evaluated with Xcalibur software (version 2.1.0). After completion of the HPTLC-MS/MS analyses, the plate was documented with a DigiStore 2 system at white light illumination in order to connect the MS, MS^2^, and MS^3^ spectra with positions (R_F_ on the plate).

## 4. Conclusions

This is the first report about: (i) comparison of chromatographic fingerprints of leaves of Japanese, Bohemian and giant knotweed; (ii) quantification of total content of monomers (flavan-3-ols) and dimers proanthocyanidins in leaves of all three knotweed species; (iii) evaluation of all three knotweed species based on comparison of B-type proanthocyanidins total peak areas; (iv) identification of all B-type proanthocyanidins from monomers to decamers and some of their gallates in all three knotweed species; (v) analyses and identification of flavan-3-ols and proanthocyanidins in leaves of Bohemian knotweed by using mass spectrometry. To the best of our knowledge this is the first report on detection of: (i) procyanidin B2 in Japanese knotweed leaves; (ii) (+)-catechin, (−)-epicatechin and procyanidin B2 in Bohemian knotweed leaves; (iii) (−)-epicatechin gallate, dimer procyanidin B1 and trimer procyanidin C1 in giant knotweed leaves. Leaves of all studied species contained (−)-epicatechin and procyanidin B2.

This study proved that our methods [13] (previously developed for the HPTLC separation of proanthocyanidins according to degree of polymerization and their identification by HPTLC-MS/MS in Japanese knotweed rhizomes) are also applicable for analyses of proanthocyanidins in other matrices like knotweed leaves. We confirmed that leaves of invasive Japanese, Bohemian and giant knotweeds are rich in proanthocyanidins and can be a potential source of proanthocyanidins, although the contents differ among the knotweeds and was the highest in giant and the lowest in Japanese knotweed. Overall leaves of Japanese, Bohemian and giant knotweed have the same chemical profiles of proanthocyanidins with respect to the degree of polymerization, while the chemical profiles of proanthocyanidins differ with respect to gallates. Giant knotweed leaves have the highest variety of gallates (from monomers to hexamers and also dimer digallate), while only monomer gallates and dimer gallates were confirmed in Japanese knotweed and monomer gallates, dimer gallates and dimer digallates were detected in leaves of the Bohemian knotweed. The profile of the Bohemian knotweed clearly shows the traits inherited from Japanese and giant knotweed from which it originated. Giant knotweed leaves and Japanese knotweed rhizomes [13] had matching qualitative chemical profiles of proanthocyanidins concerning the degree of polymerization and gallates. Within the individual species, Japanese knotweed leaves and rhizomes have equal qualitative chemical profiles of proanthocyanidins concerning the degree of polymerization, while concerning the profile of gallates rhizomes possess higher variety than leaves. HPTLC chromatographic chemical profiles of proanthocyanidins enabled differentiation among leaves of Bohemian, Japanese and giant knotweeds, as well as between Japanese knotweed leaves and rhizomes.

The qualitative data presented in this article may form the basis for further studies of the knotweeds and their secondary metabolites. Quantitative data can be a starting point for planning the possible use of knotweeds either as a new source of new biologically active compounds, or even the production of various products. This study confirmed that there is great potential for the use of these widespread plants. Since knotweeds represent both problems and opportunities, perhaps the utilization of this biomass can be a step towards solving the ecological and economic problems created by invasive knotweeds in Slovenia, Europe, and North America.

## Figures and Tables

**Table 1:** Confirmation of presence of flavan-3-ols and proanthocyanidins based on matching of the R_F_ values of standards and blue bands in tracks of STSs from leaves of Japanese (JK), Bohemian (BK) and giant (GK) knotweed on all HPTLC plates (silica gel and cellulose) developed with different developing solvents. The HPTLC silica gel plate was developed with toluene–acetone–formic acid (3:6:1, *v*/*v*) [12], while HPTLC cellulose plates were developed using the following developing solvents: water [17,18,19] and 1-propanol-water-acetic acid in different ratios (4:2:1, *v*/*v*; 20:80:1, *v*/*v*) [19]. The final confirmation of compounds was only awarded to those which were detected using both stationary phases (column “Both” in Table 1) and all developing solvents.

				HPTLC Stationary Phases											
	Silica Gel			Cellulose									Both		
				Developing Solvents											
	3:6:1 (*v*/*v*)			Water			4:2:1 (*v*/*v*)			20:80:1 (*v*/*v*)			All		
Compounds	JK	BK	GK	JK	BK	GK	JK	BK	GK	JK	BK	GK	JK	BK	GK
(+)-Catechin	+	+	+	-	+	+	-	+	+	-	+	+	-	+	+
(−)-Epicatechin	+	+	+	+	+	+	+	+	+	+	+	+	+	+	+
(−)-Catechin gallate	−	−	−	−	−	−	−	+	+	−	−	−	−	−	−
(−)-Epicatechin gallate	−	−	+	+	+	+	−	−	+	−	−	+	−	−	+
(−)-Gallocatechin	−	−	−	−	−	−	−	−	−	−	−	−	−	−	−
(−)-Epigallocatechin	−	−	−	−	−	−	−	−	−	−	−	−	−	−	−
(−)-Gallocatechin gallate	+	+	+	−	−	−	+	+	+	−	−	−	−	−	−
(−)-Epigallocatechin gallate	−	−	−	−	−	−	−	−	−	−	−	−	−	−	−
Procyanidin B1	−	−	+	−	+	+	−	−	+	−	−	+	−	−	+
Procyanidin B2	+	+	+	+	+	+	+	+	+	+	+	+	+	+	+
Procyanidin B3	−	−	−	−	−	+	−	−	−	−	−	−	−	−	−
Procyanidin C1	+	+	+	−	−	+	+	+	+	+	+	+	−	−	+

+ Identified; − Not detected.

**Table 2:** Contents of monomers and dimers in leaves of Japanese, Bohemian and giant knotweed, expressed per dry weight (DW) of the plant material, were quantified based on (−)-epicatechin and procyanidin B2 calibration curves, respectively.

Plant Organs	Knotweed	Contents of Monomers		Contents of Dimers	
		mg/100 g DW	kg/t DW	mg/100 g DW	kg/t DW
**Leaves**	Japanese	84	0.84	99	0.99
	Bohemian	139	1.39	140	1.40
	Giant	236	2.36	206	2.06

**Table 3:** Proanthocyanidins identified with HPTLC–MS/MS analyses of leaves of Japanese knotweed (**JK**), Bohemian knotweed (**BK**) and giant knotweed (**GK**). HPTLC diol F_254S_ plates were pre-developed and developed (up to 9 cm) with acetonitrile.

Compound	[M-H]^−^	[M-2H]^2−^/2	[M-3H]^3−^/3	Leaves			Rhizomes
				JK	BK	GK	JK [12,13]
Monomers	289			+	+	+	+
Monomer gallates	441			+	+	+	+
Dimers	577			+	+	+	+
Dimer gallates	729			+	+	+	+
Dimer digallates	881			−	+	+	+
Trimers	865			+	+	+	+
Trimer gallates	1017			−	−	+	+
Tetramers	1153			+	+	+	+
Tetramer gallates	1305			−	−	+	+
Pentamers	1441	720		+	+	+	+
Pentamers gallates	1593	796		−	−	+	+
Hexamers	1729	864		+	+	+	+
Hexamer gallates		940		−	−	+	+
Heptamers		1008		+	+	+	+
Octamers		1152	768	+	+	+	+
Nonamers		1297	863	+	+	+	+
Decamers		1440	960	+	+	+	+

+ Identified; − Not detected.
